# The complete chloroplast genome sequence of *Vitis vinifera* × V*itis labrusca ‘Shenhua’*

**DOI:** 10.1080/23802359.2020.1855605

**Published:** 2021-01-16

**Authors:** Lipeng Zhang, Dinghan Guo, Miaomiao Liu, Feifei Dou, Yi Ren, Dongmei Li, Shiping Wang, Lei Wang, Chao Ma, Qian Zha, Ling Su, Lei Gong, Huaifeng Liu

**Affiliations:** a Department of Horticulture, College of Agriculture, Shihezi University, Shihezi, Xinjiang, China; b Xinjiang Production and Construction Corps Key Laboratory of Special Fruits and Vegetables Cultivation Physiology and Germplasm Resources Utilization, Shihezi, Xinjiang, China; c Department of Plant Science, School of Agriculture and Biology, Shanghai Jiao Tong University, Shanghai, China; d Shandong Academy of Grape, Jinan, Shandong, China; e Shanghai Academy of Agriculture Sciences, Shanghai, China

**Keywords:** *Vitis vinifera* × *Vitis labrusca ‘Shenhua’*, chloroplast genome, Illumina sequencing, phylogenetic analysis

## Abstract

*Vitis vinifera* × *Vitis labrusca ‘Shenhua’* is a tetraploid grape, a Franco-american species. This study first published the complete chloroplast genome of *Vitis vinifera* × *Vitis labrusca ‘Shenhua’* was assembled. The chloroplast genome is 160928 bp in length, including a large single copy region (89,148 bp), a small single-copy region (19,072 bp) and a pair of inverted repeats of 26,354 bp. The chloroplast genome encodes 133 genes, comprising 88 CDSs, 37 tRNA genes and 8 rRNA genes. The phylogenetic tree demonstrated that *Vitis vinifera* × *Vitis labrusca ‘Shenhua’* is different from the other 16 varieties.

*Vitis vinifera* × *Vitis labrusca ‘Shenhua’* shows strong heat-resistant and disease resistance. Thus, it is widely used for breeding of heat-resistant grape varieties. It is also an excellent wine and food grape variety because of its high anthocyanin, titratable acids and soluble solids contents (Zha et al. 2020). The complete chloroplast genome of *Vitis vinifera* × *Vitis labrusca ‘Shenhua’* was assembled (GenBank: MT916288) and can be perform to phylogenetic analysis. It supplies significant information for the basic research of grape resistance breeding.

Genomic DNA was extracted from leaves of *Vitis vinifera* × *Vitis labrusca ‘Shenhua’* originated from the Zhuang-hang Experimental Station of Shanghai Academy of Agricultural Sciences (30°89’N, 121°39’W) and stored at the Center for Viticulture and Enology, Shanghai Jiao Tong University, the sample was called *‘Shenhua’*. This DNA was used to prepare 400 bp tiny fragment DNA library and then sequenced by the NovaSeq-PE150 sequencing platform (Illumina, CA, USA). 3.49 Gb clean reads were obtained in the aggregate. The complete chloroplast genome was composed by SPAdesv3.9.0 (Bankevich et al. 2012) and A5-MiSeqv20150522 (Coil et al. 2015) software. Genome functional annotation is done on the website (https://chlorobox.mpimp-golm.mpg.de/geseq.html). The complete chloroplast genome of *Vitis vinifera* (GenBank; DQ424856) serve as a reference (Jansen et al. 2006).

The length of chloroplast genome is 160,928 bp, consistsing of a large single copy region (89,148 bp), a small single-copy region 19,072 bp and a pair of inverted repeats of 26,354 bp. The chloroplast genome includes 133 single genes, including 88 protein-coding genes (CDS), 37 tRNA and 8 rRNA genes. 37.38% and 62.62% are the amounts of GC content and AT content of the grape genome. In these genes, most of them are single-copy, whereas 7 CDS (*rp123*, *rp12*, *rps7*, *rps12*, *ndhB*, *ycf2*, *ycf15*), 4 rRNAs (*rrn4.5*, *rrn-16*, *rrn5*, *rrn-23*) and 7 tRNAs (*trnL-CAA*, *trnI-CAU*, *trnV-GAC*, *trnA-UGC*, *trnI-GAU*, *trnR-ACG*, *trnN-GUU*) appear as double-copies.

A Maximum Likelihood (ML) phylogenetic tree was builded by using 16 *Vitis* species through the MEGA7 (Kumar et al. 2016) to affirm the phylogenetic position of *Vitis vinifera* × *Vitis labrusca ‘Shenhua’* within the family Vitaceae. The phylogenetic tree demonstrated that the 17 *Vitis* species are divided into two classes (Figure 1), which concordant with the former report (Xie et al. 2017). The phylogenetic tree demonstrated that *Vitis vinifera* × *Vitis labrusca ‘Shenhua’* is different from the other 16 varieties.

**Figure 1.**
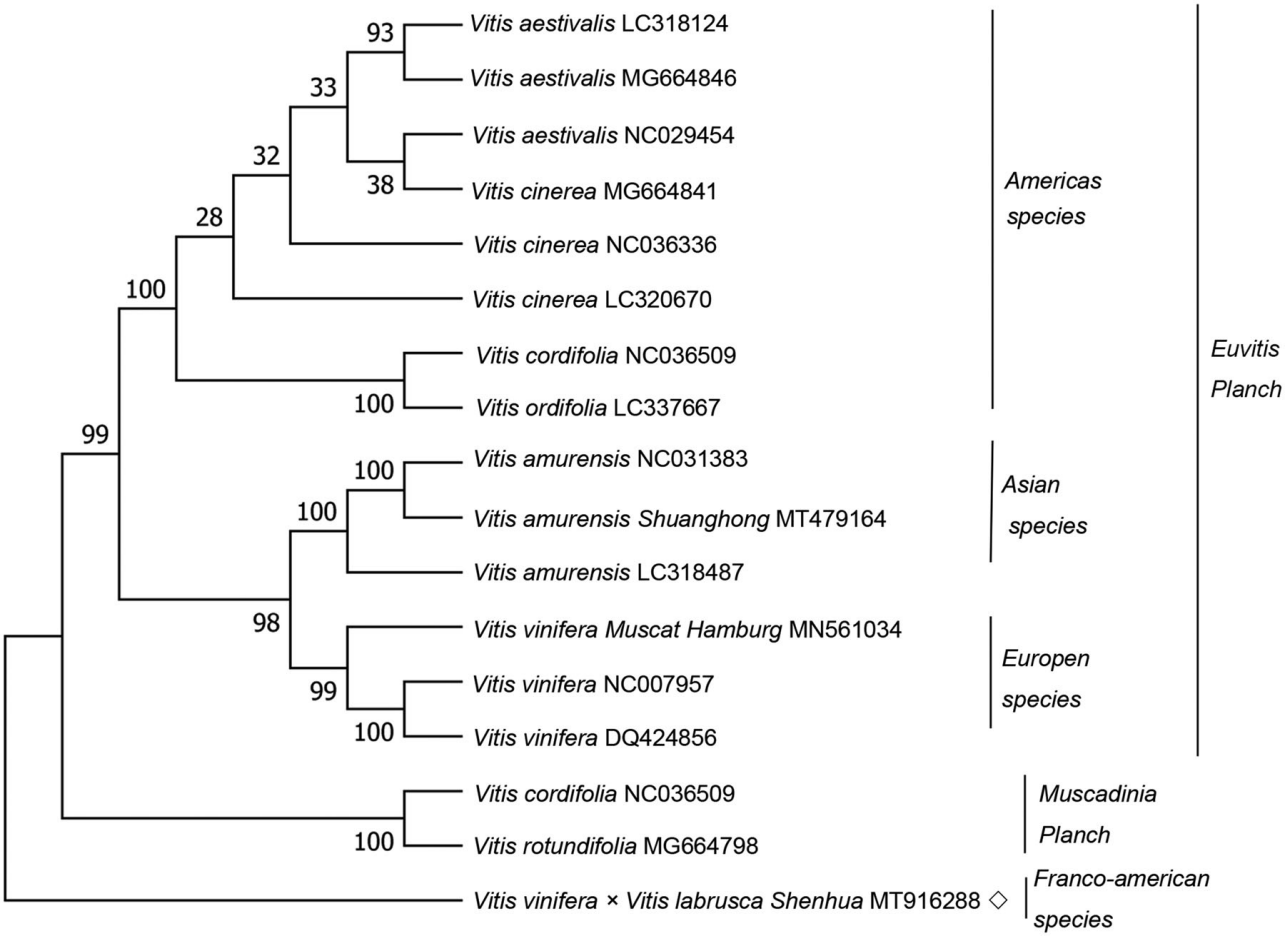
A maximum likelihood (ML) phylogenetic tree was constructed by using 17 Vitis species. The bootstrap values were based on 500 repetitions, and were shown next to the branches. ◇ indicates *Vitis* variety in this study.

## Supplementary Material

Supplemental Material

## Data Availability

The data that support the findings of this study are available in GenBank: MT916288 at https://www.ncbi.nlm.nih.gov/genbank/.
