# Supplementary figures and images for: The complete chloroplast genome sequence of Vitis vinifera × Vitis labrusca ‘Shenhua’
Source: Mitochondrial DNA B Resour. 2021 Jan 16;6(1):166–7. doi: 10.1080/23802359.2020.1855605 (PMC7832510; doi:10.1080/23802359.2020.1855605)

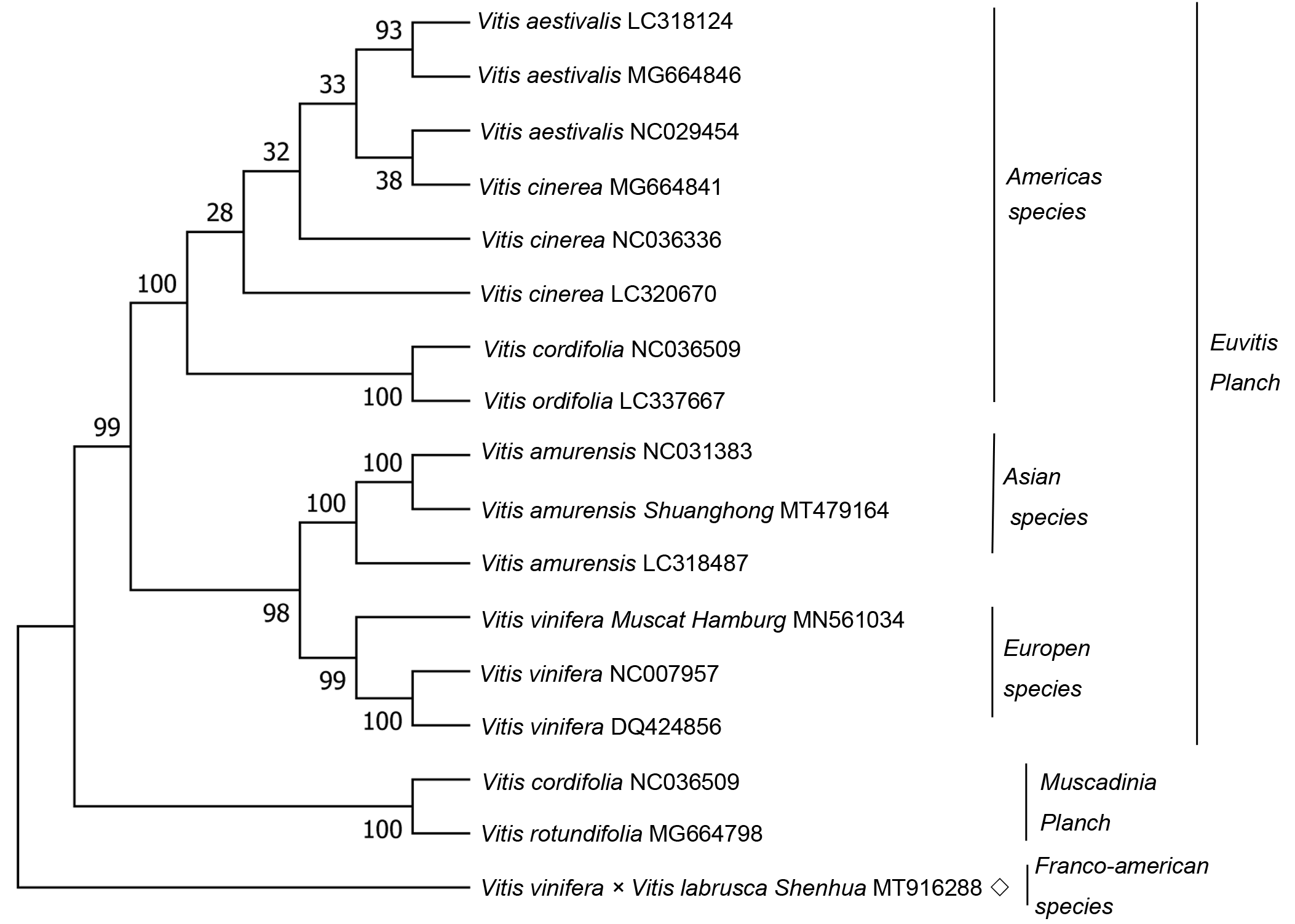

Supplement: Supplemental Material [file TMDN_A_1855605_SM9110.tif]
